# Rigorous evaluation of a pregnancy prevention program for American Indian youth and adolescents: study protocol for a randomized controlled trial

**DOI:** 10.1186/s13063-017-1842-6

**Published:** 2017-02-27

**Authors:** Lauren Tingey, Rachel Chambers, Novalene Goklish, Francene Larzelere, Angelita Lee, Rosemarie Suttle, Summer Rosenstock, Kristin Lake, Allison Barlow

**Affiliations:** 1 Johns Hopkins Center for American Indian Health, 415 N. Washington St, Baltimore, MD 21231 USA; 2 Johns Hopkins Center for American Indian Health, 308 Kuper St, Whiteriver, AZ 85941 USA

**Keywords:** Randomized controlled trials, HIV, Sexually transmitted infections, Pregnancy and childbirth, Health behavior, American Indian, Adolescent

## Abstract

**Background:**

American Indian adolescents have one of the highest rates of teen pregnancy and repeat teen births in the US. Substance use is a significant risk factor for unprotected sex, and American Indian adolescents have the highest substance use-related morbidity and mortality of any US racial group. Despite these disparities, there are no existing, evidence-based programs for pregnancy prevention that have been rigorously evaluated among American Indian teens.

**Methods:**

The proposed study is a randomized controlled trial to test the efficacy of a comprehensive sexual and reproductive health program developed in partnership with an American Indian community. Participants will be American Indians ages 11–19 and their parent or trusted adult, randomized to receive the control condition or intervention called Respecting the Circle of Life: Mind, Body and Spirit. The intervention includes eight lessons delivered to self-selected peer groups during a summer basketball camp and one lesson delivered to the youth and parent/trusted adult together within 3 months after camp. All lessons are administered by trained community health workers from the participating American Indian community. Youth and parent/trusted adult participants will complete assessments at baseline, 3, 9, 12, 24 and 36 months post-intervention completion. The primary outcome variables are sexual/reproductive health knowledge, sexual initiation, condom use self-efficacy and intent to use a condom at next sex as changed from baseline to post-intervention between intervention and control participants. Selected primary outcomes are applicable to all study participants.

**Discussion:**

Currently there are no sexual and reproductive health programs designed specifically for American Indian youth that have been rigorously evaluated and found to have an evidence base. Respecting the Circle of Life is highly innovative by incorporating lesson delivery into a summer basketball camp and involving parents or other trusted adults in curriculum administration. If found successful, it will be the first evidence-based program for teen pregnancy prevention for American Indian youth and adolescents.

**Trial Registration:**

Clinicaltrials.gov, NCT02904629. Retrospectively registered on 23 September 2016.

**Electronic supplementary material:**

The online version of this article (doi:10.1186/s13063-017-1842-6) contains supplementary material, which is available to authorized users.

## Background

American Indian (AI) adolescents have one of the highest rates of teen pregnancy of all US racial groups [1]. In 2013, American Indian/Alaska Native (AI/AN) females 15 to 19 years old had the third highest teen birth rate in the US (31 per 1000 compared with 27 per 1000 nationally), and in 2010, they had the highest prevalence of repeat teen births (21.6% compared with 20.9%, 20.4% and 14.8% among Hispanic, Black and White females, respectively) [1–3]. Nearly half (41%) of AI females begin childbearing in adolescence and within their lifetime bear twice as many children as the general US population [2, 3]. Compared with US All Races, AI adolescents are more likely to have ever had sex (69% vs. 47%), had sex for the first time before age 13 (11% vs. 6%), had sex with four or more persons during their lifetime (22% vs. 15%) and drank alcohol or used drugs prior to sex (32% vs. 22%) [4–6].

Substance use is one of the most significant risk factors for unprotected sex, and AI teens have the highest drug-related morbidity and mortality of any US racial group [7–10]. Compared to all other US adolescents, in 2009, AI youth were more likely to engage in past-month binge drinking (30% vs. 24%), marijuana use (32% vs. 21%) and cocaine use (6% vs. 3%), and nearly three times as many reported lifetime methamphetamine use (11% vs. 4%) [11].

Therefore, one tribal community in partnership with long-term academic research collaborators developed a comprehensive sexual and reproductive health program for AI adolescents. In the participating tribal community, unintended and teen pregnancies are high and many children are born to unwed mothers. These challenges are perpetuated by the absence of sexual and reproductive health education in the local school curriculum and the lack of evidence-based teen pregnancy prevention programs targeting AI adolescents.

“Respecting the Circle of Life: Mind, Body and Spirit” was adapted in 2010 by the tribal-academic partners from an evidence-based intervention (EBI) for the prevention of HIV/AIDS called “Focus on Youth (FOY) + Informed Parents and Children Together (ImPACT).” FOY consists of eight lessons delivered weekly by pairs of adult interventionists from the community to self-selected same-sex peer-groups in community centers [12]. ImPACT is a separate, one-time lesson delivered to the teen and parent together by an adult interventionist after the initial eight lessons; it is comprised of a DVD and workbook. FOY + ImPACT first targeted low-income, urban African-American youth and has a track-record of successful cross-cultural replication in various international contexts [13–16].

FOY + ImPACT was selected for adaptation by tribal-academic partners because of its targeted age group (adolescents), skills-focused curricula, theoretical underpinnings promoting positive protective factors and capacity for delivery by trained community members. Further, FOY + ImPACT’s core elements and key characteristics matched community-identified needs for this program including: (1) a lack of sexual health education in schools; (2) the role of substance use as a primary risk factor for early sexual and substance use initiation and unprotected sex; (3) multi-level curriculum administration including through peer groups and with parents or other trusted adults.

To adapt FOY + ImPACT, tribal-academic partners utilized a community-based participatory research approach, which included leadership from a Community Advisory Board (CAB), continuous engagement with tribal stakeholders and qualitative research comprising 18 focus groups with teens and parents from the participating tribal community. Key themes emerged from the qualitative data collection namely limited knowledge of and use of sexual risk reduction strategies, low condom use self-efficacy, poor negotiation and communication skills between adolescent sexual partners, the influence of peers on sexual decision-making (especially among males) and infrequent parent-child communication related to sexual health in families [17].

Reflecting on these themes, we made the following adaptations to the FOY + ImPACT curriculum: (1) enhance content around reproductive anatomy, sexual and reproductive health information, pregnancy prevention strategies and applied skills training; (2) teach proper condom and contraceptive use and community- and youth-centered strategies for overcoming access barriers; (3) build in comprehensive substance use prevention components teaching peer refusal and pro-peer relations; (4) reflect the importance of social pressure on males and self-esteem and connectedness on females for sexual decision making [17]; (5) modify all content to reflect the local language, include familiar characters, and add culturally relevant examples and scenarios; (6) reproduce the ImPACT DVD with local actors and testimonials, include traditional Native storytelling, and add emphasis on the balance among physical, spiritual and emotional health.

Several changes were also made to curriculum delivery structure [18]. Qualitative findings indicated the FOY peer-group component should be collapsed from 8 weekly to 8 daily sessions and implemented during a summer basketball camp [17]. Additionally, offering the ImPACT youth-parent lesson to “non-traditional” parents and/or caregivers that play an important role in youths’ lives was determined appropriate and necessary. The original architect of FOY + ImPACT, Dr. Bonita Stanton, endorsed all changes and adaptations as feasible and highly practical.

In 2011 and 2012, we conducted a pilot trial of the Respecting the Circle of Life (RCL) peer-group component (8 lessons taught during summer basketball camp) without the additional youth-parent lesson [18, 19]. Immediately post-camp, RCL participants had significantly greater condom use self-efficacy, intention to use condoms at next sex, HIV/STI prevention and transmission knowledge, partner negotiation skills related to alcohol and drug use during sex, and belief in the protective effects of condoms and abstinence [19]. RCL participants were also more likely to talk with a family member or other adult about HIV/AIDS [19]. Regarding the theoretical constructs underpinning the RCL intervention, all positive protective factors, including self-efficacy (ability to engage in behaviors to reduce risk for STIs/HIV), response efficacy (ability of the protective behavior to actually reduce risk, i.e., efficacy of condoms to prevent infection and pregnancy) and response cost (negative impacts of engaging in the protective behavior), were significantly improved among RCL participants [19].

Improvements in condom use self-efficacy, HIV/STI prevention and transmission knowledge, belief in the protective effects of condoms, talking with a family member or other adult about HIV/AIDS, and response efficacy were sustained at 6 months follow-up [19]. Changes in condom use self-efficacy and response efficacy were sustained through 12 months [19]. All other impacts attenuated at 12 months, which is consistent with previous trials of the FOY peer-group component [14–16, 20]. In an effort to extend intervention impacts past 6 months, previous research groups added the ImPACT youth-parent component and found both existing and additional intervention impacts (including substance use initiation and subsequent substance use) sustained through 24 months [21, 22].

Thus, the aim of this trial is to evaluate the effect of the RCL intervention, including both peer-group (8 lessons) and youth-parent components (1 lesson), on knowledge, attitudes and behaviors associated with unprotected sex and unintended pregnancy among an exclusive sample of AI adolescents. Utilizing a randomized controlled trial (RCT) design, we will test whether the RCL intervention affects well-established intermediate outcomes and risky sexual behaviors that predict a long-term impact on the incidence of teen pregnancy [23].

## Methods/Design

### Trial Design

The study is a randomized controlled superiority trial with two parallel groups and primary endpoints of condom use self-efficacy, condom use intention, sexual and reproductive health knowledge, and delayed sexual initiation 9 months post-intervention. Block randomization will be performed with a 1:1 allocation.

### Participants

Youth participants will be of AI ethnicity, ages 11 to 19 and have primary residence on or near the participating tribal reservation community. Parent/trusted adult participants will be chosen by the youth and be the parent/legal guardian of the participating youth or another family member or trusted adult over the age of 18. If the youth choses an adult who is not the legal guardian, the adult must be approved by the legal guardian. Participants who are unable to fully participate in the evaluation assessments or intervention components will be excluded (see Table 1 for Inclusion and Exclusion criteria). All participants will complete informed consent to participate; youth under age 18 will have parent/guardian consent.

**Table 1:** Inclusion and exclusion criteria

Inclusion criteria		
	Youth	-Self-identified American Indian -11-19 years old -Primary residence on or near the reservation -Written informed consent (parent/guardian consent if 11–17 years old)
	Parent/trusted adult	-Parent, legal guardian or trusted adult -18 years old or older -Primary residence on or near the reservation -Written informed consent
Exclusion criteria		
	Youth	-Unwilling to be randomized -Unable to complete intervention because of cognitive limitations -Unable to complete evaluation (i.e., planned move, residential treatment, etc.)
	Parent/trusted adult	-Unwilling to be randomized -Unable to complete intervention because of cognitive limitations -Unable to complete evaluation (i.e., planned move, residential treatment, etc.)

We will use non-probability sampling to recruit participants through public postings in community gathering spots (i.e., supermarket, daycares, fitness center, etc.), through public service announcements on the local radio, by print advertising in the local newspaper, during public gatherings (i.e., health fairs, parades, etc.) and through local schools. We will recruit three cohorts of participants and host three summer basketball camps (2016, 2017 and 2018).

### Intervention: Respecting the Circle of Life: Mind, Body and Spirit (RCL)

RCL consists of a peer-group (adapted FOY lessons) and a youth-parent component (adapted ImPACT lesson). The peer-group component consists of eight lessons, each lasting ~120 min, delivered by two trained AI community health workers to self-selected same-sex peer groups of 8–12 AI youth. The eight lessons are delivered once per day during the 8-day summer basketball camp. The youth-parent component is one educational lesson lasting 90–120 min delivered within 3 months after the end of camp, to the youth and parent/trusted adult, together with an AI community health worker.

RCL focuses on: (1) extrinsic rewards, by teaching decision-making related to communication and negotiation skills around sex, as well as information regarding birth control and condom use, and (2) intrinsic rewards, by emphasizing values clarification and goal setting. Peer-group instruction incorporates discussion, lectures, games, role playing and storytelling. RCL content covers: (1) reproductive and sexual health education; (2) instruction about condom use, contraception, STIs, safe sex practices and pregnancy prevention; (3) partner communication and negotiation skills for sex and substance use; (4) peer refusal for unwanted sex and substance use; (5) peer norms related to delaying sexual initiation; and (6) linkages to community resources. Youth-parent instruction incorporates a DVD and skills-based activities focused on: (1) information about sexual and reproductive health; (2) promoting family values pertaining to sex and substance use; (2) techniques for youth-parent communication and effective parental monitoring; (3) how to support youth condom and contraceptive use; and﻿ (4) decision-making pertaining to sex.

### Control Program

The control program is identical in structure and delivery mode to RCL and consists of peer-group and youth-parent components. The control program does not include any content pertaining to sexual and reproductive health, to ensure no overlap. Lesson content includes: (1) knowledge about food labels and general nutrition; (2) information about different types of physical activity; (3) activities to encourage youth to eat healthy; and (4) relaxation techniques. Implementing a control program identical in structure but different in content will ensure observed impacts on key outcomes can be attributed to the content of the RCL program.

### Randomization

Participants will be individually randomized to receive the intervention or control program. Participants will be assigned to either program through a stratified block randomization sequence using Stata 14 [24] to ensure a 1:1 allocation of study conditions occurs within each gender and age sub-group (11–12, 13–15 and 16–19). Since both the RCL and control program include a youth-parent component, siblings will be randomized to the same group.

### Data Collection Time Points

**Fig. 1:**
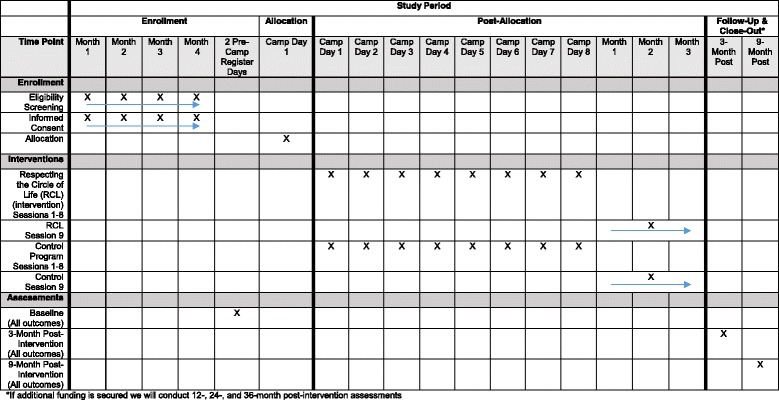
SPIRIT Figure

The flow of participants from recruitment through the end of the study is illustrated in the SPIRIT Study Figure (Fig. 1). Youth and parent participants will be assessed at three time points: baseline, 3 months and 9 months post-intervention completion. Due to the nature of the intervention, neither participants not interventionists can be blinded to group allocation. Therefore, all follow-up assessments are done by ﻿research staff (independent evaluators) that are blinded. If unblinding occurs, another independent evaluator will be brought in to re-establish blindness. Youth and parent participants will be given a gift card after completing each assessment totaling $100 for youth and $60 for parents, respectively.

All data collection will be administered via ACASI (Audio Computer Assisted Self-Interview) technology on a laptop or tablet, which includes an audio component for participants with literacy challenges. This method has been shown to decrease response bias in AI communities, especially around sensitive topics [25]. The youth baseline assessment will be administered during two pre-camp registration days prior to randomization; the parent-trusted adult baseline will be administered prior to camp at the time of consent. All youth and parent/trusted adult follow-up assessments will be delivered in participant’s homes, at school or another location of their choosing.

### Outcomes

Youth participants will complete the Youth Health Risk Behavioral Inventory (YHRBI) developed by Stanton and colleagues to evaluate the FOY + ImPACT program [26]. The YHRBI assesses demographics; knowledge, intentions and past experience regarding risk and protective behaviors; sexual perceptions; and behaviors including sexual initiation, alcohol/drug use during sex, condom use and contraceptive use. The reliability of this assessment has been established in previous evaluations [26], including our pilot trial in which the majority of sub-scales had Cronbach’s alpha values ≥0.70 [18].

Youth and parent/trusted adult participants will also complete the Parental Monitoring Scale and Parent Adolescent Communication Scale, both successfully used in previous trials of FOY + ImPACT [27, 28]. The Parental Monitoring Scale assesses perceptions of parental supervision, and the Parent Adolescent Communication Scale measures family-based communication. During the formative work previously discussed, we adapted all three scales with the participating tribal community to ensure use of local terminology, age and culturally appropriate examples, relevant theoretical constructs, clarity and flow.?>

#### Primary Outcome Measures

The primary outcome variables are sexual/reproductive health knowledge, sexual initiation, condom use self-efficacy and intent to use a condom at next sex as changed from baseline to post-intervention between intervention and control participants. These variables were chosen because they are applicable to all study participants.

#### Secondary Outcome Measures

Secondary outcome variables will be examined among sexually active participants including condom use at last sex, number of sexual partners, sex without a condom in the last 3 months and sex without contraception in the last 3 months. It is expected that 20-25% of study participants will be sexually active. The impact evaluation will also have exploratory outcome variables including parental engagement (communication and monitoring), youth substance use and substance use just prior to and/or during sex.

### Sample Size Calculation and Statistical Analysis

Our goal is to recruit 567 youth (and their parent/trusted adult) balanced within two age groups (11–12, 13–15 and 16–19), for a total of 1134 participants (567 youth and 567 parents/trusted adults). We expect a maximum attrition rate of 25%, based on past rates in similar evaluations [18, 19, 29, 30].

Sample size estimates were based on the variables of condom use self-efficacy and sexual initiation because these confirmatory research questions apply to all study participants, and prior studies have shown them to be associated with reductions in risky sexual behavior, teen pregnancy and STI incidence [23]. We hypothesize that the RCL intervention will increase condom use self-efficacy in the intervention group compared to the control group and that the intervention group will have a lower proportion of participants who initiate sex, compared to controls, at study end line.

The condom use self-efficacy scale ranges from 1–5. Our pilot trial [18] provided data on the condom use self-efficacy score among the control group (mean = 2.6, SD = 1.0), degree of intra-team correlation (“k” = 0.12) and potential loss to follow-up over 9 months (20% average reduction in team size, i.e., from 9 down to 7.2 youths). Although this trial will utilize individual randomization, the intervention will be carried out with teams of 8–12 participants each, which will require accounting for intra-team correlation.

We will use the average of two follow-up measures of condom use self-efficacy per participant (intra-measure correlation = 0.7) to reduce error in outcome measurement [31]. To achieve 90% power to detect a theoretically meaningful between-group difference of 0.47 points on this scale over 9 months post-intervention, ~32 teams per study arm will be required. Thus, we will enroll and randomize a total of *n* = 284 participants per study arm (32 teams/arm *9 participants/team), for a total sample size of *n* = 567 participants. We have reduced the significance level used in sample size calculations to 0.0125 to account for multiplicity corrections during analyses. This same sample size will be sufficient to detect a 19.7% between-group difference, with 80% power and 0.0125 significance level, in the percent of participants who report ever having had sex. This estimate is conservative based on our past retention rates and accounts for up to 25% loss to follow-up, while adjusting for the intra-team correlation.

This sample size will also be able to detect meaningful differences between the intervention and control groups with at least 80% power, given the assumptions listed above: 6.7% between-group difference in reproductive/sexual health knowledge score [estimating a mean score (SD) of 76% (16%) in the control group] and 20.5% between-group difference in intention to use a condom at next sex (estimating 55% in the control group).

Study hypotheses will be initially tested under an “intent-to-treat” model in which data are analyzed according to randomization assignment. We will then conduct “completer analyses” on those subjects finishing at least two-thirds of the 9 intervention sessions. Intervention impact will be evaluated by comparing primary study outcomes between intervention and control groups across the three time points: baseline, 3 and 9 months post-intervention. Initially, summary scores of outcomes will be stratified by participation in the intervention versus control groups and by time point and compared using chi-square tests (for binary or categorical outcomes), t-tests and analysis of variance (for continuous outcomes). The equivalence of the two study groups at baseline will be compared across a range of background variables. Generalized linear mixed effects models (GLMMs) accounting for within-group correlation across longitudinal data will then be applied.

Outcomes will be modeled as a function of group assignment and time since intervention. The association between outcomes and time will be explored visually using scatterplots and lowess smoothers. Restricted cubic splines will be added to reflect deviations from linearity. GLMM with random intercepts for peer group and study participant will be used to account for the hierarchical structure of the data: peer group, participants nested within peer group and assessments nested within study participants. If siblings enter the study together during the same wave, they will be randomized to the same study group (intervention or control) to limit contamination. In this case, analyses will be adjusted to take into account intra-sibling correlation as well. Missing data will be handled as follows: (1) document reason(s) for missing data to inform model development; (2) assess treatment dropouts to do intent-to-treat analysis; (3) conduct sensitivity analysis to compare inferences that are based on different plausible reasons for missingness [32, 33].

Current funding for this evaluation supports data collection through 9 months post-intervention. If additional funding is secured, we will extend data collection to 12, 24 and 36-months post-intervention and collect medical chart data to track incidence of STIs and pregnancy. If additional funding is secured, gift cards in amounts commensurate with the time required to complete additional follow-up assessments will be given.

### Methods Against Bias

Separate cadres of research staff will be hired and trained as RCL or control facilitators and independent evaluators. Facilitators will be responsible for delivering either the RCL or control program and will not participate in any aspect of the evaluation. Independent evaluators will conduct screening for inclusion and exclusion criteria, obtain informed consent, enroll eligible youth and parents/trusted adults, and administer all evaluations to youth and parents. Independent evaluators will not participate in any aspect of RCL or control program delivery. All research staff are members of the participating tribal community and employed/trained by the academic research partner. Research staff complete a background check and are extensively trained in human subject’s research prior to any interaction with participants or study data.

To ensure the RCL and control programs are implemented in a comparable way, all facilitators will receive extensive training in their respective program and must pass a proficiency examination with a score ≥85% prior to delivery. In addition, fidelity monitoring of RCL and control program implementation will be performed on ≥10% of all sessions conducted. Sessions will be randomly selected and observed in person, audio recorded or video recorded. Staff performing fidelity monitoring will complete a feedback form and review it with the RCL or control facilitator. Additional training will be conducted as necessary.

To reduce potential for contamination across study groups, RCL and control programs are delivered in separate camp locations. Youth in each group participate in basketball play only with other youth receiving the same program (RCL or control). All staff members, including basketball coaches, are instructed not to discuss the content of either program outside of the classroom. Siblings will also be randomized to the same group (RCL or control) to avoid contamination within families.

Data quality and maintenance of confidentiality are continuously monitored by the principal investigator. A unique participant ID code will be used to administer and track all data collection, stored separately from data collection tools and locked in a secure room with limited access by authorized individuals. Data will be stored in a database that is password protected and located on a secure server. Only the personal identifier of participants’ date of birth will be included in the database. Any electronic documents that link IDs to identifying information (i.e., names/signatures on consent forms and logs) will be stored in accordance with the academic partner’s data security guidance. Transfer or storage of data on portable devices (e.g., laptops, flashdrives) will be encrypted and accessible only to study personnel.

### Safety and Ethical Issues

This trial has received approval from the funding agency and relevant Indian Health Service and University research review boards. Any necessary protocol modifications will be submitted as amendments for review to these relevant parties. The trial and manuscript were approved by the participating tribal community’s governing bodies. Independent evaluators will obtain informed written consent from all adult participants and assent and parental permission from all minor participants (<18 years of age). Independent evaluators will explain the study including procedures, risks and benefits associated with participating, and the rights and responsibilities of study participants. Staff will encourage participants to ask questions and provide copies of the consent form. Participants will be informed that they can stop study participation at any time. The Principal investigator has obtained a Certificate of Confidentiality from the Department of Health and Human Services, which states researchers cannot be forced to disclose information that may identify participants, even by court subpoena, in any federal, state or local civil, criminal, administrative, legislative or other proceedings. Any serious adverse event reported or detected during the trial will be documented and reported to the relevant research review boards within 24 h. There is no Data and Safety Monitoring Board for this trial. [Note: a completed SPIRIT checklist (Additional file 1) was submitted along with the manuscript to the journal].

## Discussion

The RCL intervention is ground-breaking in that it successfully promotes concentric circles of support for adolescents’ positive behavior change at individual, peer, family and community levels. Intergenerational intervention has special import to AI communities as family is generally valued as the nexus of strength for individuals and been shown to yield great influence on adolescents’ behavioral choices [34–36]. Also, historical and present-day trauma have eroded traditional family structures (i.e., poverty and overcrowded households, single parent homes, high residential mobility) and contributed to intergenerational cycles of teen pregnancy. Native families experiencing these stressors may rely on a combination of immediate and extended family members as well as close friends for caretaking [37]. Native parents and communities are eager for prevention interventions that strengthen entire family networks; thus, incorporation of parents or trusted adults in the RCL program is socially and culturally congruent for AI youth and families and holds promise for reducing accumulated risk across generations [37].

Currently there are no interventions for the prevention of STIs, HIV and teen pregnancy designed specifically for American Indian youth that have been rigorously evaluated and found to have an evidence-base. RCL is highly innovative as it: (1) incorporates a summer basketball camp and home visits, (2) fills health education gaps in rural, reservation-based communities, (3) targets males and females equally, both key to the prevention equation, (4) acknowledges the inextricable link between substance use and unintended pregnancy and (5) addresses intergenerational cycles of teen pregnancy in communities experiencing the repercussions of historical and modern day trauma. If RCL is found successful, it will be the first evidence-based program for teen pregnancy prevention among American Indian youth and adolescents.

### Trial status

Youth and parents began to enter the trial in May 2016. Recruitment is still open.

## Additional file

Additional file 1: SPIRIT Checklist. (DOC 123 kb)

## Abbreviations

ACASI: Audio Computer Assisted Self-Interview
AI: American Indian
AI/AN: American Indian/Alaska Native
CAB: Community Advisory Board
EBI: Evidence-based intervention
FOY: Focus on Youth
GLMM: Generalized Linear Mixed Effects Models
ImPACT: Informed Parents and Children Together
RCL: Respecting the Circle of Life
RCT: Randomized Controlled Trial
YHRBI: Youth Health Risk Behavioral Inventory
